# Emerging Blood-Based Biomarkers for Predicting Response to Checkpoint Immunotherapy in Non-Small-Cell Lung Cancer

**DOI:** 10.3389/fimmu.2020.603157

**Published:** 2020-10-16

**Authors:** Shumin Li, Chengyan Zhang, Guanchao Pang, Pingli Wang

**Affiliations:** Department of Respiratory and Critical Care Medicine, The Second Affiliated Hospital of Zhejiang University School of Medicine, Hangzhou, China

**Keywords:** immunotherapy, biomarker, programmed death ligand 1, liquid biopsy, non-small-cell lung cancer

## Abstract

Immune checkpoint inhibitors (ICIs) have brought impressive clinical benefits in a variety of malignancies over the past years, which dramatically revolutionized the cancer treatment paradigm. Monotherapy or in combination with chemotherapy of ICIs targeting programmed death 1/programmed death ligand 1 (PD-L1) has emerged as an alternative treatment for patients with advanced non-small-cell lung cancer (NSCLC). However, constrained by primary or acquired resistance, most patients obtain limited benefits from ICIs and occasionally suffer from severe immune-related adverse events. Moreover, owing to the complexity of the tumor microenvironment and the technical limitations, clinical application of PD-L1 and tumor mutation burden as biomarkers shows many deficiencies. Thus, additional predictive biomarkers are required to further advance the precision of proper patient selection, avoiding the exposure of potential non-responders to unnecessary immunotoxicity. Nowadays, an increasing number of investigations are focusing on peripheral blood as a noninvasive alternative to tissue biopsy in predicting and monitoring treatment outcomes. Herein, we summarize the emerging blood-based biomarkers that could predict the clinical response to checkpoint immunotherapy, specifically in patients with NSCLC.

## Introduction

Immunotherapy has revolutionized the strategy for the treatment of cancer and it aims to activate the immune system to identify and destroy cancer cells. Patients with multiple malignancies, such as melanoma, renal carcinoma, and lung cancer, significantly benefit from immune checkpoint inhibitors (ICIs) (1). Regardless of their use as first- or second-line treatment, ample evidence from several clinical trials demonstrated that ICIs are superior to platinum-based chemotherapy in the treatment of advanced non-small-cell lung cancer (NSCLC) (2–5). Unfortunately, only approximately 20%–40% of patients with advanced NSCLC obtained durable clinical benefits from anti-programmed death (ligand) 1 [anti-PD-(L)1] therapies (2–5). In other words, in most cases, patients have primary or acquired resistance to checkpoint immunotherapy (6, 7). Additionally, non-responders not only suffer from risk of severe immune-related adverse events, but also meaninglessly afford the high cost of anti-PD-(L)1 drug. Therefore, early identification of potential responders versus non-responders is of great clinical significance. In recent years, research studies aim to identify appropriate biomarkers that could stratify patients into potential responders and non-responders prior to the initiation of immunotherapy, as well as monitor clinical response in real time during treatment.

In clinical practice, PD-L1 expression assessed by immunohistochemistry on tumor samples obtained through biopsy is the most extensively used predictive biomarker. It is approved by the Food and Drug Administration as a companion or complementary diagnostic assay for the administration of ICIs in patients with NSCLC (8). Across multiple clinical trials performed in advanced NSCLC patients treated with anti-PD-(L)1 antibody, high pretreatment PD-L1 expression corresponded to superior clinical outcomes. However, the application of tumor PD-L1 expression as a predictive biomarker remains defective and controversial, as numerous patients with PD-L1-negative tumors may also respond to anti-PD-(L)1 therapy. Furthermore, immunohistochemistry analysis of PD-L1 expression remains technically and biologically limited, namely due to the existence of intra-tumoral heterogeneity, different expression levels between primary and metastatic lesions, and a lack of standardization in the detection antibodies and scoring systems (9, 10). Moreover, archival tissue specimens collected in the past may not reflect current PD-L1 expression levels (11), particularly in patients receiving chemotherapy, radiotherapy, or anti-angiogenic therapy after biopsy (12, 13).

Beyond PD-L1 expression, tumor mutation burden (TMB) was also included into the National Comprehensive Cancer Network guidelines recently as an emerging predictor of response to ICIs. TMB is the total number of non-synonymous somatic mutations per megabase in the coding region of the cancer genome (14). In previous clinical studies conducted in patients with NSCLC, TMB showed value in identifying responders to ICIs, with data supporting that higher TMB values correlated with improved progression-free survival (PFS), superior overall response rate (ORR), and better durable clinical benefits. For example, the Checkmate-227 and Checkmate-568 studies demonstrated that TMB ≥ 10 muts/Mb could predict clinical benefits of nivolumab plus ipilimumab in patients with NSCLC, regardless of PD-L1 expression (15, 16). However, the latest data from the Keynote-021 and Keynote-189 studies yielded inconsistent evidence, indicating that the predictive value of TMB in combined immunotherapy is uncertain (17–19). Similar with PD-L1 expression, TMB is also characterized by a few limitations, such as the high cost of whole-exome sequencing, long result turnaround time, and complicated analytical process. Moreover, techniques used to evaluate TMB level are not standardized, and the predictive threshold differ widely across next-generation sequencing platforms. In addition, multiple preanalytical and analytical factors, such as sample collection, fixation methodology, and sequencing depth, may influence the TMB value (20).

Tissue samples are required to detect the aforementioned biomarkers, while tumor biopsy is invasive, occasionally infeasible, and cannot be repeatedly performed to monitor early disease response in most patients. Furthermore, biopsy specimens are difficult to accurately reflect the overall condition of the tumor due to the existence of intra-tumoral heterogeneity (21). Hence, currently approved tissue biomarkers are characterized by these practical limitations. In contrast, peripheral blood is a non-invasive, low-risk, and convenient source for repetitive sampling. Therefore, researchers show increasing interest in developing blood-based biomarkers for patient selection and treatment monitoring. Furthermore, peripheral blood could provide a holistic insight into the host immune status, which is one of the decisive factors for the efficacy of cancer immunotherapy (22). Numerous studies have explored the potential blood-based predictors of response to anti-PD-(L)1 therapy, such as circulating immune cells, circulating tumor cells (CTCs), and cytokines. In this systematic review, we summarize the findings of recent studies utilizing various components of peripheral blood to predict the efficacy of anti-PD-(L)1 therapy in patients with NSCLC.

## Routine Peripheral Blood Biomarkers

Owing to the easy accessibility and low cost, numerous studies have investigated the predictive value of blood routine parameters in tumor immunotherapy. In multivariable analysis of NSCLC patients treated with nivolumab, the baseline absolute lymphocyte count ≥ 1,000/μl and absolute neutrophil count (ANC) ≤ 7,500/μl were significantly and independently correlated with both prolonged PFS [hazard ratio (HR) = 0.55, p = 0.04 and HR = 3.97, p = 0.001, respectively] and better overall survival (OS) (HR = 0.36, p = 0.03 and HR = 3.46, p = 0.03, respectively) (23). However, another study observed that a high absolute lymphocyte count did not correspond to favorable OS after anti-PD-1 treatment; meanwhile, an increased ANC only correlated with shorter OS (HR = 1.86, p = 0.02) (24). These data suggested that the absolute lymphocyte count or ANC alone cannot adequately predict clinical outcomes and efficacy of response to checkpoint immunotherapy in patients with NSCLC.

An elevated neutrophil-to-lymphocyte ratio (NLR) indicates the chronic inflammation status, which could be used to reflect the immune status of patients with different malignancies (25). In this regard, several studies investigated the negative prognostic value of high NLR in NSCLC patients receiving immunotherapy. Bagley et al. reported that NLR ≥ 5 at baseline was significantly correlated with worse OS (HR = 2.07, p = 0.002) and PFS (HR = 1.43, p = 0.04) (26). Subsequently, data from Suh et al. also supported that NLR ≥ 5 at 6 weeks post anti-PD-1 treatment was associated with poor PFS (HR = 15.09, p < 0.001) and OS (HR = 3.82, p = 0.003) (27). Furthermore, Cao et al. carried out a meta-analysis including 14 retrospective studies with 1,225 NSCLC patients treated with nivolumab, further confirming that a baseline NLR ≥ 5 was associated with inferior PFS (HR = 1.73, p < 0.05) and OS (HR = 1.76, p < 0.05) (28).

Researchers proposed that the derived NLR [dNLR, ANC/(white blood cell−ANC)] may be more relevant to clinical outcomes than the NLR, as the former also takes monocytes and other granulocyte subpopulations into account. In advanced NSCLC patients treated with anti-PD-(L)1 therapy, an association between baseline dNLR > 3 and shorter OS was revealed (HR = 1.70, p < 0.001). In addition, baseline dNLR together with lactate dehydrogenase (LDH) constituted a lung immune prognostic index, which effectively distinguished three groups of patients with different survival outcomes (median OS: 16.5 vs. 10 vs. 4.8 months; p < 0.01) (29). In another study, the advanced lung cancer inflammation index (ALI) was calculated as body mass index × albumin/NLR, and high systemic inflammation was suspected in patients with a low ALI score. Multivariate analyses indicated that pretreatment ALI < 18 was independently related to poor PFS and higher risk of early progression [odds ratio (OR) = 2.76, p = 0.002] in NSCLC patients receiving nivolumab (30).

Investigation of the absolute eosinophil count, absolute monocytic count, platelet-to-lymphocyte ratio, and lymphocyte-to-monocyte ratio as predictors of response to ICIs also attracted considerable interest over the past years. Clinical studies showed that pretreatment absolute eosinophil count ≥ 150/μl and absolute monocytic count ≥ 630/μl negatively impact PFS (HR = 0.53, p = 0.02 and HR = 1.50, p = 0.02, respectively) in NSCLC patients treated with anti-PD-1 agent (23, 24). In addition, platelet-to-lymphocyte ratio ≥ 169 at 6 weeks correlated with longer OS (HR = 1.56, p = 0.002) (27). Except for the aforementioned parameters, Sekine and colleagues proposed and validated that an increased lymphocyte-to-monocyte ratio was significantly associated with higher ORR (50.0% vs. 20.0%; p = 0.015) and prolonged PFS (not reached vs. 3.1 months; p = 0.0092) (31). Early changes in lymphocyte-to-monocyte ratio could be further explored as an effective marker to evaluate whether anti-PD-1 therapy should be continued.

Finally, several exploratory studies provided a clue that baseline level of serum LDH may contribute to the identification of patients with NSCLC gaining better survival benefits from immunotherapy. Researchers evaluated the LDH level in 94 NSCLC patients treated with ICIs, and discovered that baseline LDH < 400 was related to improved OS (HR = 0.45, p = 0.01) (32). Further studies involving large-scale prospective cohorts may facilitate the progress in confirming the function of elevated LDH in NSCLC and its potential value in predicting efficacy.

## Circulating Immune Cell Subsets

### Dynamic Changes in T Lymphocytes

Continuous advancements in the application of high-throughput technologies in multi-parametric flow cytometry, single-cell sequencing, and mass cytometry (CyTOF) make it possible to monitor the dynamic changes in different circulating immune cell subtypes from peripheral blood collected during cancer immunotherapy. Previous studies have provided evidence that utilizing the positive surface expression of PD-1 enables the identification of tumor-specific T cells in multiple malignancies, and PD-1^+^ CD8^+^ T cells could also be detected in peripheral blood (33–35). The proliferation response of circulating PD-1^+^ CD8^+^ T cells is more likely to be tumor-specific, as non-tumor-specific CD8^+^ T cells do not present an increase in the frequency of Ki-67^+^ cells after immunotherapy (36, 37). In this regard, researchers speculated that the anti-tumor cytotoxicity of T cells infiltrated in the tumor microenvironment could be reflected by the reinvigoration status of circulating PD-1^+^ CD8^+^ T cells during anti-PD-(L)1 immunotherapy. Moreover, circulating PD-1^+^ CD8^+^ T cells may be an important determinant of response to ICIs.

Several research studies have paid more attention to the predictive value of the proliferation response of circulating PD-1^+^ CD8^+^ T cells. Huang et al. analyzed the clonal overlap between tumor-infiltrating CD8^+^ T cells and circulating CD8^+^ T cells in melanoma patients receiving PD-1-targeted immunotherapy with pembrolizumab. The results showed that top-ranked CD8^+^ T-cell clones in peripheral blood are also present in tumor tissues, all of which were CD38^+^ HLA-DR^+^ and mostly Ki-67^+^ PD-1^+^ (38). The frequency of Ki-67^+^ cells among PD-1^+^ CD8^+^ T cells 3 weeks post treatment/baseline tumor burden (Ki-67/TB) ratio was applied to predict the clinical efficacy. They found that Ki-67/TB ratio > 1.94 was correlated with increased ORR (p = 0.03), prolonged PFS (p = 0.03), and OS (p = 0.004) in the discovery cohort. However, data from the validation cohort did not show strong association between the Ki-67/TB ratio and survival outcomes (38). Subsequently, Kamphorst et al. observed that the proliferation of peripheral PD-1^+^ CD8^+^ T cells within 4 weeks of anti-PD-(L)1 therapy in patients with NSCLC was associated with good clinical response (37). These proliferating CD8^+^ T cells presented an effector-like phenotype (HLA-DR^+^ CD38^+^ BCL-2^low^) and exhibited increased expression of positive costimulatory molecule CD28, suggesting a vital role in response to anti-PD-(L)1 therapy (37). Similarly, results from Kim et al. supported the idea that early proliferation of peripheral PD-1^+^ CD8^+^ T cells could predict response to anti-PD-1 therapy. Fold changes in the frequency of Ki-67^+^ cells among PD-1^+^ CD8^+^ T cells 7 days after treatment (Ki-67_D7/D0_) were used to evaluate the rate of early proliferation in three independent cohorts. In the validation NSCLC cohort, a Ki-67_D7/D0_ ≥ 2.8 was closely associated with superior durable clinical benefits (p = 0.001), as well as prolonged PFS (p = 0.002) and OS (p = 0.037) (36). Moreover, this study also indicated that Ki-67_D7/D0_ was more reliable in predicting non-responders, as its negative predictive value ranged 85%–94%. Taken together, the above independent studies highlighted the predictive value of circulating Ki-67^+^ PD-1^+^ CD8^+^ T cells. Hence, monitoring this T-cell subpopulation during treatment may provide informative data on outcomes. However, further studies are warranted to confirm their practicality as predictors of efficacy.

Conversely, Simoni et al. demonstrated that PD-1 was also expressed on the surface of non-tumor-specific bystander tumor-infiltrating CD8^+^ T cells, and CD39 could be a more straightforward marker for distinguishing tumor-specific T cells (39). The expansion of the circulating CD39^+^ T-cell subpopulation might be an early sign of cytotoxic anti-tumor-specific responses and could be exploited for the development of promising biomarkers. In addition to CD8^+^ T cells, CD4^+^ T cells also play an important role in determining the efficacy of anti-PD-(L)1 therapy. A recent study, which included NSCLC and renal cell carcinoma patients receiving nivolumab and pembrolizumab, indicated that the differential peripheral change between responders and non-responders was observed in the TIM-3^+^ T-cell subset. The increase in the frequency of TIM-3^+^ cells either among CD4^+^ T cells or CD8^+^ T cells was independently correlated with lower PFS (12-month PFS: 0% vs. 81.5%; p < 0.001 and 20.8% vs. 85.7%; p = 0.002, respectively) (40). Noteworthy, this study was limited by its small sample size, and its main methodological weakness was that blood was collected only 12 weeks after treatment. Further studies are necessary to evaluate these blood parameters at earlier sampling time points.

### Baseline Level of T Lymphocytes

Compared with dynamic biomarkers, the advantage of baseline biomarkers is that they can help predict the response to immunotherapy before the initiation of treatment. Whereas the therapeutic response displays features of a critical state transition involving complex immunological processes; therefore, it is notoriously difficult to predict the response far in advance (41–43). Despite these limitations of static biomarkers, researchers have consistently explored the potential value of baseline immune cells as predictive biomarkers in patients treated with anti-PD-(L)1 agent.

Manjarrez-Orduno et al. discovered that melanoma and non-squamous NSCLC patients with increased transcription of inflammatory genes in tumor tissues always presented a high central memory T cell to effector T cell ratio in peripheral blood. In NSCLC patients treated with nivolumab, a high central memory/effector T cell ratio showed a strong correlation with higher tumor PD-L1 expression and prolonged PFS (91 vs. 215 days) (44). The existence of terminally differentiated T cells might explain this un-expected inverse correlation between the number of effector T cells and response to ICIs. These results are concordant with another contemporaneous research study performed in patients with melanoma, which demonstrated that peripheral blood mononuclear cells (PBMCs) obtained from responders exhibited lower frequencies of effector memory CD4^+^ T cells and naïve CD8^+^ T cells, and higher frequencies of central memory CD8^+^ T cells (45). Additionally, Kim and colleagues found that lower frequency of effector memory (CCR7^−^ CD45RA^−^) CD8^+^ T cells and a higher frequency of severely exhausted population (TIGIT^+^ cells among PD-1^+^ CD8^+^ T cells) at baseline were associated with inferior clinical outcomes and increased risk of hyperprogression disease (HPD) (46). The advantage of this study was the analysis of HPD data from a large population of patients with NSCLC. Prospective validation of these biomarkers in the future will properly guide the selection of patients who will obtain clinical benefits from anti-PD-(L)1 therapy.

Several studies claimed that clinical response to anti-PD-(L)1 therapy requires the existence of functional systemic CD4 immunity. Zuazo et al. reported that NSCLC patients with a higher proportion of highly differentiated (CD27^−^ CD28^−^) CD4^+^ T cells are more prone to superior clinical outcomes (47). Further analysis revealed that these highly differentiated CD4^+^ T cells were mainly composed of non-exhausted memory (CD45RA^−^ CD62L^+/−^) CD4 cells, which significantly affect T cell proliferation response during immunotherapy (47). Subsequently, this research team conducted a prospective study in 70 NSCLC patients treated with ICIs, indicating that HPD was closely associated with dysfunctional CD4 immunity and an increased number of peripheral CD28^−^ CD4^+^ T cells (48). Julia et al. also highlighted the importance of functional CD4 T cell immunity for anti-tumor response, and they observed higher baseline proportion of central memory CD4^+^ T cells in responders (40). Closely consistent with these studies, Kagamu et al. reported that patients showing response to nivolumab generally presented a higher frequency of CD62L^low^ CD4^+^ T cells (p < 0.0001) at baseline. In contrast, CCR7^−^ CD4^+^ T cells did not show a significant difference between responders and non-responders (49). Furthermore, CyTOF analysis revealed that the majority of CD62L^low^ CD4^+^ T cells corresponded to double-negative CD27^−^ CD28^−^ T cells, and was significantly correlated with the classical Th1 (CXCR3^+^ CCR4^−^ CCR6^−^) subpopulation (p < 0.0001) (49). Regretfully, the sample size of these studies was relatively small, and the concluded potential biomarkers remain to be further investigated.

Regulatory T (Treg) cells constitute a special immunosuppressive subset, which could promote immunosuppression and tolerance in patients with tumor. It has been speculated that a low percentage of Treg cells might contribute to the efficacy of ICI therapy. However, researchers found that the frequency of CD127^−^ CD25^+^ Treg cells did not show a significant difference between responders and non-responders, either at baseline or post-treatment (45). These results agree well with the observation of Huang et al. that there was no significant correlation between Ki-67^+^ proliferating Treg cells and clinical outcomes (38). Consistent with the initial hypothesis, Kagamu et al. reported that the baseline proportion of CD25^+^ FOXP3^+^ cells among total CD4^+^ T cells was significantly higher in non-responders (p = 0.034) (49). Based on the ratio of CD62L^low^ T cells to CD25^+^ FOXP3^+^ CD4^+^ T cells, they further developed a formula for the prediction of non-responders with 85.7% sensitivity and 100% specificity (49). In contrast, several studies found that higher Treg cell counts at baseline was associated with better prognosis in patients receiving anti-PD-(L)1 therapy (50, 51). Researchers evaluated the frequency of lectin-type oxidized LDL receptor 1 (LOX-1^+^) polymorphonuclear myeloid-derived suppressor cells (PMN-MDSCs) in NSCLC patients treated with nivolumab. They demonstrated that the ratio of Treg cells to PMN-MDSCs could predict clinical outcomes in both discovery and validation cohorts (51). These findings warrant further validation in large cohort studies, and novel strategies should be devised to detect the varying immunosuppressive activity of Treg cells in individual patients. Future exploration of Treg cells as a predictive biomarker should consider the differences in the immunosuppressive function of Treg cells, rather than focusing merely on the Treg cell count.

### T Cell Receptor Repertoire

Activation of the host immune system against cancer cells requires the recognition of neoantigen peptides by clonally proliferating T cell receptors (TCRs) (52). The clonality and diversity of TCRs can be assessed by deep sequencing of the complementary-determinant region 3, which depends on the random variable-(diversity)-joining recombination (53). Studies in various tumors have investigated whether peripheral TCR repertoire analysis could serve as a predictive biomarker of response to immunotherapy (54–58). However, the predictive value of the peripheral TCR repertoire remains controversial, likely because the existence of non-tumor-specific TCRs in PBMCs diluted tumor neoantigen-specific TCRs (59).

Several studies confirmed the observation that exhausted neoantigen-specific T cells can be reinvigorated by ICIs, and their subsequent clonal expansion was associated with the anti-tumor immune response (60, 61). In addition, numerous studies have indicated that the PD-1^+^ CD8^+^ phenotype represents an exhausted T cell subset, and it could be exploited to monitor dynamic changes in neoantigen-specific cytotoxic T cells in multiple malignancies (33–35). Recently, Han et al. collected PBMCs from NSCLC patients treated with ICIs, and specifically investigated the TCR repertoire in PD-1^+^ CD8^+^ exhausted T cells. Patients with higher pretreatment TCR diversity and increased post-treatment TCR clonality in sorted peripheral PD-1^+^ CD8^+^ T cells, were more likely to obtain superior survival outcomes (62). This study is of great significance as it marks the first attempt to evaluate the TCR repertoire specifically in PD-1^+^ CD8^+^ exhausted T cells instead of total T cells. In addition, the independent validation study also enhances the credibility of these conclusions. Overall, more evidence is needed before utilizing the TCR repertoire as a predictive biomarker for immunotherapy in clinical practice.

### MDSCs

MDSCs are groups of highly heterogeneous cells derived from immature myeloid progenitors with potent immunosuppressive properties (63). According to morphological characteristics and phenotypic analysis, human MDSCs are usually divided into PMN-MDSCs and monocytic MDSCs (M-MDSCs). In human peripheral blood, CD11b^+^ CD14^−^ CD15^+^ or CD66b^+^ are generally used to characterize PMN-MDSCs, while CD11b^+^ HLA-DR^−/low^ CD14^+^ CD15^−^ cells are equivalent to M-MDSCs (64). It is generally believed that PMN-MDSCs can be separated from peripheral blood by standard Ficoll density gradient centrifugation. However, a recent study suggested that PMN-MDSCs and activated CD15^+^ neutrophils without suppressive activity can simultaneously appear in PBMC layer after Ficoll gradient separation (65). Fortunately, a study reported that LOX-1 was specifically expressed on immunosuppressive PMN-MDSCs, but not on neutrophils in peripheral blood (66). Thus, the combination of gradient centrifugation with LOX-1 expression helps distinguish circulating PMN-MDSCs from neutrophils in patients with cancer.

MDSCs participate in the regulation of anti-tumor immunity through various immunosuppressive mechanisms, such as inducing nitric oxide synthase and arginase (67), producing reactive oxygen species (68), increasing Treg cells (69) and directly inhibiting the proliferation of T lymphocytes (70). Based on the immunosuppressive functions of MDSCs, several studies explored the specific role of MDSCs in predicting clinical efficacy of PD-1-targeting antibody in patients with NSCLC. A study performed in such patients reported that, after the first dose of nivolumab, the proportion of LOX-1^+^ PMN-MDSCs was significantly higher in non-responders than responders (51). Patients with a higher post-treatment Treg cells to LOX-1^+^ PMN-MDSCs ratio showed superior PFS in both discovery and validation cohorts (51). Another study analyzed blood immune parameters in patients with metastatic NSCLC before and during treatment of nivolumab. They noted that high expression of TIM-3 on lymphoid cells and early accumulation of (Lin^−^ CD33^+^ CD14^+^ CD15^−^ HLA-DR^−^) M-MDSC associate with resistance to PD-1 blockade. Moreover, TIM-3^+^ lymphoid cells and galectin-9 positive M-MDSC impeded the secretion ability of CD8^+^ T cells and reduced the efficacy of PD-1-targeting antibody (71). Passaro et al. reported that NSCLC patients with high baseline level of PMN-MDSCs and a low CD8 to PMN-MDSC ratio had significantly improved response to immunotherapy (p = 0.02) (72).

These findings suggest that MDSCs have potential in predicting response to anti-PD-(L)1 therapy in patients with NSCLC. However, the underlying mechanisms and interactions involved in this process still require further investigation. Besides, unlike other immunosuppressive cells, various MDSC subsets lack uniform definitions, and the results are inconsistent in different studies. Therefore, more prospective studies yielding solid and definitive evidence are required prior to applying these findings for the guidance of clinical decision.

### Monocytes and Natural Killer Cells

Besides T cells and MDSCs, researchers using high-dimensional CyTOF and bioinformatics analysis deeply characterized the peripheral immune cell subsets of melanoma patients treated with immunotherapy. Krieg et al. described that baseline classical monocytes (CD14^+^ CD16^−^ HLA-DR^hi^) was a promising immune predictor of response. The results from the independent validation cohort using conventional flow cytometry also confirmed that classical monocytes may aid in guiding treatment decisions (45). However, to date, there are no study investigating whether circulating monocytes could serve as a predictor of response in NSCLC patients treated with anti-PD-(L)1 therapy.

Regarding natural killer (NK) cells, several studies explored the potential relationship between circulating NK cells and response to ICIs. In NSCLC patients treated with ICIs, Cho et al. discovered that the overall activity of NK cells and their count were significantly higher in responders compared with non-responders (73). However, this preliminary study included only nine patients; thus, a large-scale study will be needed in the future to confirm these results. In patients with NSCLC, Mazzaschi et al. observed that the absolute number of circulating CD56^+^ NK cells at baseline resulted in a two-fold higher change in responders compared with non-responders (p < 0.01). During the administration of nivolumab, the circulating NK cell count progressively increased in responders, while remained stable or slightly decreased in non-responders (74). Conversely, Ottonello et al. recently reported that a relatively high frequency of baseline NK cells was closely related to poor OS and PFS in NSCLC patients treated with nivolumab (75). These results indicate that the specific function of NK cells in immunotherapy and their potential as biomarkers for predicting response to ICIs require further investigation.

These findings emphasized to the importance of the peripheral immune status in predicting response to anti-PD-(L)1 therapy. Nevertheless, all these divergent investigational biomarkers require further confirmation by prospectively designed studies with large cohorts. We summarized the studies exploring peripheral immune cells as predictive biomarkers of response to ICIs in patients with NSCLC (**Table 1**).

**Table 1:** Investigational peripheral immune cell biomarkers of response to ICIs in NSCLC.

Biomarker	Cancer type	No. of patients	Peripheral findings associated with clinical response	Reference
Ki-67^+^ PD-1^+^ CD8^+^ T cells	NSCLC	29	Increased proliferative response in PD-1^+^ CD8^+^ T cells within 4 weeks of treatment associated with PR or SD	Kamphorst et al. (37)
Ki-67_D7/D0_	NSCLC TET	79 31	Higher fold change of proliferative response in PD-1^+^ CD8^+^ T cells at first-week of treatment associated with DCB and prolonged PFS	Kim et al. (36)
TIM-3^+^ CD4/CD8^+^ T cells	NSCLC RCC	18 7	Increased frequency of TIM-3^+^ cells among CD4^+^ or CD8^+^ T cells correlated with poor PFS	Julia et al. (40)
CM/Eff T cell ratio	NSCLC Melanoma	62 43	Higher CM/Eff T cell ratio associated with increased tumor inflammatory profile and prolonged PFS	Manjarrez-Orduno et al. (44)
Effector memory CD8^+^ T cells TIGIT^+^ PD-1^+^ CD8^+^ T cells	NSCLC	263	Lower frequency of effector memory cells among CD8^+^ T cells associated with HPD, poor PFS and OS Higher frequency of TIGIT^+^ cells among PD-1^+^ CD8^+^ T cells associated with HPD, poor PFS and OS	Kim et al. (46)
CD27^−^ CD28^−^ CD4^+^ T cells	NSCLC	51	Higher baseline frequency of highly differentiated CD4^+^ T cells associated with prolonged PFS	Zuazo et al. (47)
(%CD62L^low^ CD4^+^ T cells)^2^/(%CD25^+^ FOXP3^+^ CD4^+^ T cells) ratio	NSCLC	126	Higher baseline prediction formula value associated with prolonged PFS and OS	Kagamu et al. (49)
(%Treg cells)/(%LOX-1^+^ PMN-MDSCs) ratio	NSCLC	63	Higher (%Treg cells)/(%LOX-1^+^ PMN-MDSCs) ratio associated with prolonged PFS	Kim et al. (51)
TCR diversity in PD-1^+^ CD8^+^ T cells	NSCLC	40	Higher baseline TCR diversity in PD-1^+^ CD8^+^ T cells and increased TCR clonality after treatment associated with prolonged PFS	Han et al. (62)
Lin^−^ CD33^+^ CD14^+^ CD15^−^ HLA-DR^−^ M-MDSC	NSCLC	61	Higher frequency of M-MDSC associated with poor OS	Limagne et al. (71)
Good Immunological asset (PMN-MDSC ≥ 6 cell/μl, ANC < 5,840/μl, AEC > 90/μl, NLR < 3)	NSCLC	53	Good Immunological asset associated with prolonged PFS and OS	Passaro et al. (72)
NK cells	NSCLC	9	Higher frequency and overall activity of NK cells associated with PR or SD	Cho et al. (73)
CD56^+^ NK cells	NSCLC	31	Increased number of NK cells associated with prolonged survival outcomes	Mazzaschi et al. (74)
NK cells	NSCLC	74	Higher frequency of NK cells at baseline associated with shorter OS and PFS	Ottonello et al. (75)

ICI, immune checkpoint inhibitor; NSCLC, non-small-cell lung cancer; PD-1, programmed death 1; PR, partial response; SD, stable disease; TET, thymic epithelial tumor; DCB, durable clinical benefit; PFS, progression-free survival; RCC, renal cell carcinoma; TIM-3, T-cell immunoglobulin mucin 3; CM, central memory; Eff, Effector; TIGIT, T cell immunoreceptor with Ig and ITIM domains; HPD, hyperprogression disease; OS, overall survival; Treg, regulatory T; LOX-1, lectin-type oxidized LDL receptor 1; PMN-MDSC, polymorphonuclear myeloid-derived suppressor cell; TCR, T cell receptor; M-MDSC, monocytic MDSC; ANC, absolute neutrophil count; AEC, absolute eosinophil count; NLR, neutrophil-to-lymphocyte ratio. NK, natural killer.

## CTCs

CTCs derived from the primary tumor and/or metastatic lesion are dispersed into the bloodstream *via* intravasation, reflecting the genetic and epigenetic variations in tumor tissue. Expression of PD-L1 on CTCs has been extensively demonstrated, and CTCs have emerged as a readily obtainable source for the evaluation of tumor evolution (76–79). According to the available data, PD-L1 expression on NSCLC-derived CTCs and the concordance rate of PD-L1 expression between CTCs and tumor tissue vary markedly in different studies (78, 80, 81). Nonetheless, multiple studies have demonstrated the prognostic value of CTCs in patients with NSCLC, and these studies indicated that dynamic changes in the number of PD-L1^+^ CTCs may help monitor response to treatment (82–85).

Several studies evaluated the expression of PD-L1 on NSCLC-derived CTCs to investigate its predictive value of selecting patients for immunotherapy. Firstly, Nicolazzo et al. longitudinally evaluated the presence of PD-L1^+^ CTCs in metastatic NSCLC patients treated with nivolumab. They found that, after 6 months of treatment, all patients with PD-L1^+^ CTCs experienced disease progression (76). Subsequently, Guibert and colleagues evaluated the expression of PD-L1 on CTCs among 96 patients with NSCLC at the initiation of treatment and time of progression. CTCs were detected in baseline blood samples in 93% of patients, and 83% of those expressed PD-L1 on ≥ 1% of CTCs. In terms of clinical outcomes, responders had lower baseline CTC counts (p < 0.0001) compared with non-responders. A high number of CTCs at baseline (> 30/7.5 ml) was significantly associated with worse clinical outcomes (OS: HR = 2.37, p = 0.0088; PFS: HR = 2.44, p = 0.0004). The presence of pre-treatment PD-L1^+^ CTCs was not significantly correlated with survival outcomes. Nevertheless, patients with higher frequency of baseline PD-L1^+^ CTCs (≥ 1%) were more likely to be non-responders (p = 0.04) (78). Similarly, another research investigated the correlation between PD-L1^+^ CTCs and PFS in 17 patients treated with anti-PD-1 therapy. The results indicated that ≥ 2 PD-L1^+^ CTCs per ml of blood at baseline was not a predictor of response to immunotherapy (86). Overall, to date, the possibility of utilizing PD-L1^+^ CTCs as a predictive biomarker has not been solidly demonstrated in patients with NSCLC.

Besides the analysis of PD-L1^+^ CTCs, a prospective exploratory cohort study (including 104 NSCLC patients receiving ICIs) investigated the predictive value of CTCs and tumor-derived extracellular vesicles. Data showed that the presence of CTCs prior to or during treatment was an independent predictor for the lack of durable response, and it was associated with shorter PFS and OS. Elevated level of tumor-derived extracellular vesicles were correlated with shorter survival, but not with the response rate (87).

Limitations of the aforementioned studies include small research cohorts, non-uniform methodology for the capture of CTCs, use of different antibodies for the staining of PD-L1, and lack of clear cut-off criteria for the definition of positive PD-L1 expression. Multicentral studies are needed to ascertain whether CTCs could serve as a predictive biomarker.

## Soluble Serum-Based Biomarkers and Cytokines

### Soluble PD-L1

Multiple research studies have demonstrated that both PD-1 and PD-L1 have soluble forms (sPD-1 and sPD-L1) in peripheral blood, and their increasing levels measured by enzyme-linked immunosorbent assay may correlate with the response to immunotherapy (88, 89). Current studies have suggested that lower levels of sPD-L1 may correlate with longer survival in several malignancies (90, 91). Zhou et al. reported that high pretreatment level of sPD-L1 in melanoma patients treated with ICIs was associated with an increased risk of progressive disease (92). However, increased post-treatment level of sPD-1 was associated with favorable clinical responses (92). Consistent with these results, a prospective study involving 39 NSCLC patients treated with PD-1-targeting antibodies also found that higher sPD-L1 level at baseline was related to a shorter OS (93). Furthermore, among patients treated with nivolumab, the ORR was higher in the low sPD-L1 group versus the high sPD-L1 group (59% vs. 25%, p = 0.0069). In addition, another single-center study including 43 NSCLC patients treated with nivolumab yielded similar results (94). Thus, baseline sPD-L1 may represent an immunosuppressive status and indicate poor response to ICIs. However, the underling mechanisms are not fully elucidated.

Recently, a case-control study proposed composite criteria (sCombo) corresponding to sPD-1 and sPD-L1 positivity for the prediction of immune response in individual patients. In the nivolumab group, baseline sCombo positivity was negatively related to PFS (HR = 2.66, p = 0.02) but not OS. Notably, increased or stable sPD-1 levels after two cycles of treatment with nivolumab was correlated with prolonged PFS (HR = 0.49, p = 0.004) and OS (HR = 0.39, p = 0.002) (95). In conclusion, sPD-L1 may represent a novel biomarker for guiding patient selection and predicting clinical outcomes. However, the lack of standardization of measurement and a consistent threshold is the major limitation for the application of sPD-L1 to clinical practice.

### Granzyme B

Granzyme B is a serine protease secreted by NK cells and cytotoxic CD8^+^ T cells, which is involved in mediating target cell apoptosis (96). Preclinical models showed that granzyme B activity can be evaluated through dedicated positron emission tomography imaging, and that tumors with a high signal for granzyme B uptake showed good response to ICIs (97, 98). Furthermore, a clinical study evaluated the concentration of soluble granzyme B in the peripheral blood of NSCLC patients treated with nivolumab. They reported that responders had significantly higher concentration of soluble granzyme B than non-responders at initiation of treatment with nivolumab (p = 0.039) (94). This may reflect an activated and efficient CD8^+^ cytotoxic immune response, known to be associated with better response to ICIs. During treatment, patients with a stable or decreased concentration of soluble granzyme B had advantages in ORR, OS, and PFS (94). A possible explanation is that the increase in the concentration of granzyme B reflects an expanding, but ineffective T-cell response leading to T-cell exhaustion.

### Indoleamine 2,3-Dioxygenase

Indoleamine 2,3-dioxygenase (IDO) is a key enzyme responsible for catalyzing the conversion of essential amino acid 1-tryptophan to the main metabolite kynurenine. Moreover, it promotes cancer cell survival through enhanced suppression of immunity (99). Growing preclinical evidence indicates that an increase in IDO activity is involved in resistance to ICIs, and IDO activity may serve as a predictor of immunotherapeutic efficacy (100). The serum kynurenine/tryptophan ratio was measured by high-performance liquid chromatography-tandem mass spectrometry to assess baseline IDO activity. The IDO activity was negatively associated with PFS and OS in advanced NSCLC patients treated with second-line nivolumab (101). These preliminary results suggested that the serum kynurenine/tryptophan ratio may guide the identification of NSCLC patients with intrinsic resistance to anti-PD-1 agents.

### Interleukin-6 and Interleukin-8

Various studies have reported that soluble cytokines influence the efficacy of ICIs. For example, Sanmamed et al. demonstrated that early changes in serum interleukin-8 (IL-8) level reflect and predict response to anti-PD-1 therapy in patients with metastatic melanoma and NSCLC. They designed a validation cohort of 19 NSCLC patients receiving anti-PD-1 agents, and they found that responders had significantly decreased levels of serum IL-8 at the best response moment, whereas non-responders presented opposite changes. in patients with NSCLC, an early decrease in serum IL-8 level was associated with longer OS (p = 0.015) (102). In addition, this study showed that serum IL-8 level could discern the appearance of pseudoprogression. Moreover, Kang et al. reported that serum interleukin-6 level at baseline could be used to predict the clinical efficacy of anti-PD-(L)1 therapy in patients with NSCLC. Patients with low interleukin-6 level (< 13.1 pg/ml) at baseline presented significantly superior PFS (6.3 vs. 1.9 months; p < 0.001) and OS (not reached vs. 7.4 months; p < 0.001) than those with high interleukin-6 level (103). Studies with larger cohorts are warranted to validate these results.

### Exosomes

Exosomes are extracellular vesicles secreted by various cells (including cancer cells), and contain DNA, RNA, and proteins (104). A study evaluated the PD-L1 mRNA expression in circulating exosomes to monitor the response to PD-1-targeting antibodies in patients with melanoma or NSCLC. The data showed that, after treatment, PD-L1 mRNA expression in exosomes significantly decreased in responders, remained unchanged in those with stable disease, and significantly increased in patieints with progressive disease (105). This study demonstrated that dynamic measurement of PD-L1 expression in circulating exosomes is feasible and might provide useful information regarding response to treatment with ICIs. Future exploration in larger cohorts of patients are required, as well as a comparison of PD-L1 expression in paired tissue and circulating exosomes.

The levels of soluble proteins and cytokines can be easily determined, providing an automated, highly sensitive, accurate, and straightforward approach to simultaneously analyzing multiple samples. Most of the aforementioned studies were exploratory, and further studies are required to verify the efficacy of these biomarkers in patients with NSCLC.

## Conclusion and Future Perspectives

The clinical exploration of peripheral blood biomarkers for immunotherapy is important and rapidly developing due to its safety and less invasive nature. In this review, we covered various potential blood-based biomarkers, such as peripheral T lymphocytes, TCR repertoire, MDSCs, CTCs, and soluble proteins. Different assays and platforms were used to monitor peripheral immune status in multiple clinical studies, therefore, demonstrating the potential of these biomarkers in predicting the efficacy of ICIs. Nevertheless, most of the available results are preliminary, so the potential biomarkers in these studies cannot be implemented into routine clinical practice until they are validated in further large-scale prospective clinical trials. Furthermore, more clinical trials should be designed to explore differences in the application of the potential biomarkers alone or in combination, and to standardize thresholds for the guidance of clinical decision making. Despite the inherent challenges, peripheral blood-based biomarkers remain attractive tools for personalized clinical management of immunotherapy for patients with NSCLC.

## Author Contributions

SL wrote the manuscript. CZ and GP provided expertise and advice. PW designed and supervised the project. All authors contributed to the article and approved the submitted version.

## Funding

This study was supported by the General Project (U1609220 and 81470212) from the National Natural Science Foundation of China and the Key Research and Development Project (2020C03027) from the Department of Science and Technology of Zhejiang Province.

## Conflict of Interest

The authors declare that the research was conducted in the absence of any commercial or financial relationships that could be construed as a potential conflict of interest.
